# Accelerated 3D T_2_w‐imaging of the prostate with 1‐millimeter isotropic resolution in less than 3 minutes

**DOI:** 10.1002/mrm.27764

**Published:** 2019-04-21

**Authors:** Rohini Vidya Shankar, Elisa Roccia, Gastao Cruz, Radhouene Neji, René Botnar, Davide Prezzi, Vicky Goh, Claudia Prieto, Isabel Dregely

**Affiliations:** ^1^ School of Biomedical Engineering and Imaging Sciences King's College London London United Kingdom; ^2^ MR Research Collaborations, Siemens Healthcare Limited Frimley United Kingdom; ^3^ Department of Radiology Guy's and St Thomas' Hospitals NHS Foundation Trust London United Kingdom

**Keywords:** 3D prostate imaging, fast imaging, isotropic resolution, T_2_‐weighted MRI, TV‐SENSE

## Abstract

**Purpose:**

To achieve 3D T_2_w imaging of the prostate with 1‐mm isotropic resolution in less than 3 min.

**Methods:**

We devised and implemented a 3D T_2_‐prepared multishot balanced steady state free precession (T_2_prep‐bSSFP) acquisition sequence with a variable density undersampled trajectory combined with a total variation regularized iterative SENSE (TV‐SENSE) reconstruction. Prospectively undersampled images of the prostate (acceleration factor R = 3) were acquired in 11 healthy subjects in an institutional review board‐approved study. Image quality metrics (subjective signal‐to‐noise ratio, contrast, sharpness, and overall prostate image quality) were evaluated by 2 radiologists. Scores of the proposed accelerated sequence were compared using the Wilcoxon signed‐rank and Kruskal‐Wallis non‐parametric tests to prostate images acquired using a fully sampled 3D T_2_prep‐bSSFP acquisition, and with clinical standard 2D and 3D turbo spin echo (TSE) T_2_w acquisitions. A *P*‐value < 0.05 was considered significant.

**Results:**

The 3× accelerated 3D T_2_prep‐bSSFP images required a scan time (min:s) of 2:45, while the fully sampled 3D T_2_prep‐bSSFP and clinical standard 3D TSE images were acquired in 8:23 and 7:29, respectively. Image quality scores (contrast, sharpness, and overall prostate image quality) of the accelerated 3D T_2_prep‐bSSFP, fully sampled T_2_prep‐bSSFP, and clinical standard 3D TSE acquisitions along all 3 spatial dimensions were not significantly different (*P* > 0.05).

**Conclusion:**

3D T_2_w images of the prostate with 1‐mm isotropic resolution can be acquired in less than 3 min, with image quality that is comparable to a clinical standard 3D TSE sequence but only takes a third of the acquisition time.

## 1 INTRODUCTION

Prostate cancer is the most common cancer in men.^1^ In asymptomatic patients recommended for prostate cancer screening, elevated serum prostate specific antigen levels and/or an abnormal digital rectal examination leads to further imaging investigations, followed by invasive biopsy for histological confirmation. Whilst a screening program based on prostate specific antigen testing remains controversial, countries such as the United States offer asymptomatic men, who are 55‐69 years, the opportunity to make an individualized decision regarding prostate specific antigen testing.^2^

Imaging, in particular multiparametric MRI (mp‐MRI) has redefined how patients with suspected cancer are managed. Mp‐MRI offers the opportunity to detect and localize clinically significant prostate cancer.^3^ It also contributes to locoregional staging, surgical and radiotherapy planning,^4^, ^5^, ^6^, ^7^ and the possibility to perform a targeted biopsy using either MRI‐ultrasound fusion or direct in‐bore guidance. A typical clinical mp‐MRI protocol for prostate cancer detection consists of a 2D multislice T_2_‐weighted (T_2_w) turbo spin echo (TSE) sequence, acquired in the transversal, sagittal, and coronal orientations, a 2D transversal multislice diffusion‐weighted single‐shot echo‐planar imaging sequence, and a dynamic contrast enhanced 3D T_1_‐weighted spoiled gradient recalled sequence.^8^ However, the lack of isovolumetric voxels in 2D acquisitions has limitations for image guided biopsy, in 3D localization and image fusion, and for surgical or radiotherapy planning and image‐guided therapy.

True 3D imaging enables the visualization of the prostate in any arbitrarily angulated anatomical plane, without loss of image quality and can be acquired in reduced time as compared to separate multiplanar 2D acquisitions.^9^, ^10^, ^11^, ^12^ An isotropic high‐resolution 3D T_2_w MRI with true volumetric coverage can potentially improve the sensitivity for cancer detection, enable more precise image‐guided biopsy, and improve surgical planning or focal treatment. It can also be used to optimal benefit in emerging cutting‐edge technologies such as simultaneous positron emission tomography (PET)/MR, wherein rapid true 3D T_2_w‐MRI images can be provided for direct fusion with 3D PET. Other application areas include MR‐guided radiotherapy, on‐board systems such as MR‐LINACS (linear accelerators), and in radiotherapy planning, which require high resolution isotropic 3D images without any geometric distortion. However, conventional 3D T_2_w TSE imaging has been hampered by long acquisition times. These sequences are also highly susceptible to motion and blurring artifacts due to the long echo train sampling. Furthermore, the use of a series of 180° refocusing pulses at higher fields (3T and above) contributes to a high specific absorption rate (SAR).

Alternative techniques using T_2_‐prepared gradient echo rather than spin echo sequences have been proposed to achieve faster 3D T_2_w imaging.^13^, ^14^ The high signal‐to‐noise ratio (SNR) efficiency of fully balanced steady state free precession (bSSFP) imaging sequences makes them a popular choice for various clinical applications, as previously demonstrated in both cardiac^15^, ^16^ and prostate T_2_‐prepared MRI,^17^ and also in combination with diffusion‐prepared prostate imaging.^18^ T_2_w‐image contrast is achieved by performing segmented acquisitions interleaving the bSSFP readout with the desired magnetization preparation.^19^, ^20^ Recently, Cartesian trajectories with centric view ordering have been proposed for 3D segmented acquisitions mainly applied in paediatric and cardiac applications.^21^, ^22^, ^23^ Advantages of a centric 3D Cartesian trajectory are that (1) no re‐gridding steps are required, thus considerably simplifying the image reconstruction process, (2) the centric acquisition in the k_y_‐k_z_ plane enables immediate encoding of the magnetization prepared signal, and (3) less sensitivity to system imperfections (such as gradient delays, eddy currents, off‐resonance errors, phase corrections) as compared to non‐Cartesian acquisitions. The trajectory is also amenable to undersampling, wherein a variable density pattern is acquired using fewer shots, thus resulting in shorter scan times.

Here, we propose to combine a T_2_‐prepared^19^, ^20^ multishot 3D bSSFP sequence with variable density undersampling and total variation regularized SENSE (TV‐SENSE) reconstruction^24^ to enable fast 3D isotropic T_2_w‐imaging of the prostate in a clinically acceptable scan time. Our study involved detailed optimization of the sequence acquisition parameters, the variable density undersampling mask, and the TV‐SENSE reconstruction to reduce the scan time, while enabling high SNR, image sharpness, and tissue contrast.

## 2 METHODS

### 2.1 Sequence/acquisition optimization

The T_2_prep‐bSSFP signals depend on several intrinsic (tissue) and extrinsic (image acquisition) parameters. Therefore, image acquisition parameter values were optimized to achieve 3 main goals: (1) maximize signal intensity, (2) maximize contrast between any 2 different tissues (healthy and cancerous), and (3) maintain short scan times and insensitivity to T_1_ recovery. A simulation framework based on the extended phase graph formalism^25^ was implemented for this purpose. The T_2_prep‐bSSFP sequence was then simulated in this framework, with variation of extrinsic and intrinsic parameter values. The extrinsic parameters optimized were the repetition time (TR), bSSFP flip angle (FA), and number of segments of the bSSFP readout (N_seg_), for a range of intrinsic T_1_ and T_2_ relaxation parameters typically observed in healthy (T_2_ = 150 ms) and cancerous (T_2_ = 50 ms) prostate tissue. Variations in signal intensity and tissue contrast were simulated for the following combinations of the extrinsic parameters, (a) FA = (0:20:100)°, TR = 1600 ms, N_seg_ = 96, (b) FA = 57°, TR = (500:500:2500) ms, N_seg_ = 96, and (c) FA = 57°, TR = 1600 ms, N_seg_ = (20:20:100) segments, at T_2_‐prep durations of 0 and 90 ms. An optimal set of acquisition parameters was identified by evaluating the magnetization response for a range of different parameter values, while considering the trade‐offs between signal intensity, contrast, acquisition time, prostate T_1_, motion artifacts, and SAR limitations at 3T.

### 2.2 In vivo imaging

This study was approved by the local institutional review board. Following written informed consent, 11 healthy male subjects (*n* = 11; age = 26 ± 6 years) were scanned on a 3T PET‐MR scanner (using only the MR‐component of the Biograph mMR, Siemens Healthcare, Erlangen, Germany) using a body matrix/spine coil. MR measurements were obtained using the proposed prototype 3D segmented bSSFP readout preceded by a T_2_‐preparation module^19^, ^20^ with duration of 90 ms and time between T_2_‐preparation modules (shot‐length = TR) of 1600 ms. The adiabatic T_2_‐preparation module consists of 4 radiofrequency pulses in the sequence ($90_{x}^{\circ }-180_{y}^{\circ }-180_{y}^{\circ }-90_{-x}^{\circ }$). Imaging parameters included: transversal plane, 304 × 304 × 60 matrix, 1 × 1 × 1 mm^3^ resolution, FA = 57°, bSSFP TR/echo time (TE) = 4/2 ms, shot duration = 1600 ms, number of segments/shot N_seg_ = 96, and 14 ramp‐up pulses with linearly increasing FA to stabilize the readout magnetization.

The 3D centric CASPR trajectory consisting of Cartesian spiral‐like profile ordering^23^ was 3‐fold (3×) prospectively accelerated by a variable density undersampling of the k‐space. For comparison, clinical standard (in compliance with PI‐RADS specifications^26^) multislice 2D and 3D (SPACE) T_2_w TSE sequences were also acquired with matched imaging parameters except the following: (i) multislice 2D T_2_w TSE – 0.6 × 0.8 × 3 mm^3^ resolution, 20 slices, TR/TE = 6470/89 ms, FA = 150°, 2 averages (ii) 3D SPACE – TR/TE = 1700/101 ms, FA = 135°, 2 averages. Detailed imaging parameters for the sequences under consideration have been summarized in Table 1. The scan time (min:s) for the multislice transversal 2D T_2_w TSE acquisition was 2:16, while that for the 3D SPACE sequence was 7:29. The 3D fully sampled T_2_prep‐bSSFP sequence required a scan time of 8:23, while the 3× prospectively accelerated T_2_prep‐bSSFP acquisition required 2:45.

**Table 1:** MR acquisition parameters for the 4 sequences under consideration

Sequence	T_2_w‐TSE (multislice)	SPACE (T_2_w‐TSE)	T_2_‐prep bSSFP (fully sampled)	T_2_‐prep bSSFP (3× accelerated)
Acquisition	2D	3D	3D	3D
FOV (mm^2^)	200 × 200	199 × 199	299 × 299	299 × 299
Resolution (mm^3^)	0.6 × 0.8 × 3	1 × 1 × 1	1 × 1 × 1	1 × 1 × 1
Image matrix	320 × 256 × 20	192 × 195 × 72	304 × 304 × 60	304 × 304 × 60
TR (ms)	6470	1700	1600	1600
TE (ms)	89	101	90	90
bSSFP TR/TE (ms)	–	**–**	4.0/2.0	4.0/2.0
Flip angle (deg)	150	135	57	57
No of averages	2	2	1	1
Echo train length	25	61	–	–
Bandwidth (Hz/px)	200	592	822	822
PE direction	R ‐ L	R ‐ L	R ‐ L	R ‐ L
Scan time (min:s)	2:16	7:29	8:23	2:45

Abbreviations: SPACE, Sampling Perfection with Application optimized Contrasts using different flip angle Evolution; PE, phase encoding.

### 2.3 Image reconstruction

All undersampled T_2_prep‐bSSFP datasets were reconstructed offline in MATLAB (Mathworks, Natick, MA) using the TV‐SENSE reconstruction described in Cruz et al^24^ and Lustig et al,^27^ which involved minimizing the following cost function:

(1) $$\hat{I}=arg\;min_{I}\left\{{\left\|EI-K\right\|}_{2}^{2}+\lambda_{S}{\left\|\nabla_{s}I\right\|}_{1}\right\}$$

where, I is the image to be reconstructed, E is the SENSE encoding operator that incorporates the coil sensitivities and the Fourier transformation, K is the 3× undersampled k‐space, ∇*_s_* is the first order finite difference operator, and *λ_S_* is the total variation regularization parameter. Data consistency is achieved using the *l*_2_ norm, while the total variation operator is essentially the *l*_1_ norm operating in the image domain. The value of *λ_S_* (2e^−06^) was empirically determined to achieve a good compromise between (1) image denoising and (2) prevention of image blurring due to over‐smoothing. The coil sensitivity maps for each dataset were generated using the ESPIRiT implementation from the BART MRI toolbox.^28^

### 2.4 Evaluation of image quality

The image quality of the accelerated T_2_prep‐bSSFP images (along all 3 imaging planes: transversal, coronal, and sagittal) for each healthy volunteer was compared with the fully sampled reference T_2_prep‐bSSFP and clinical standard multislice transversal 2D T_2_w TSE and 3D SPACE sequences by 2 experienced radiologists with more than 20 and 10 years of experience in MRI interpretation, respectively. Specifically, a subjective assessment of various metrics namely the SNR, contrast, image sharpness, presence of any artifacts from acquisition (including bSSFP banding) and the TV‐SENSE reconstruction, and overall image quality in the whole prostate was conducted and scored on a (0‐4) point scale, with the scoring criteria detailed in Table 2. Each of the above metrics was assigned a combined “agreed score” by the 2 readers. The (mean ± standard deviation) scores were subsequently tabulated and compared using the Wilcoxon signed‐rank and Kruskal‐Wallis nonparametric tests to determine any statistically significant differences (*P* < 0.05) in image quality metrics between the 4 sequences under consideration.

**Table 2:** Scoring criteria used by the 2 experienced readers

Score	Description
1	Non‐diagnostic
2	Acceptable
3	Good
4	Excellent

## 3 RESULTS

### 3.1 Sequence/acquisition optimization

**Figure 1:**
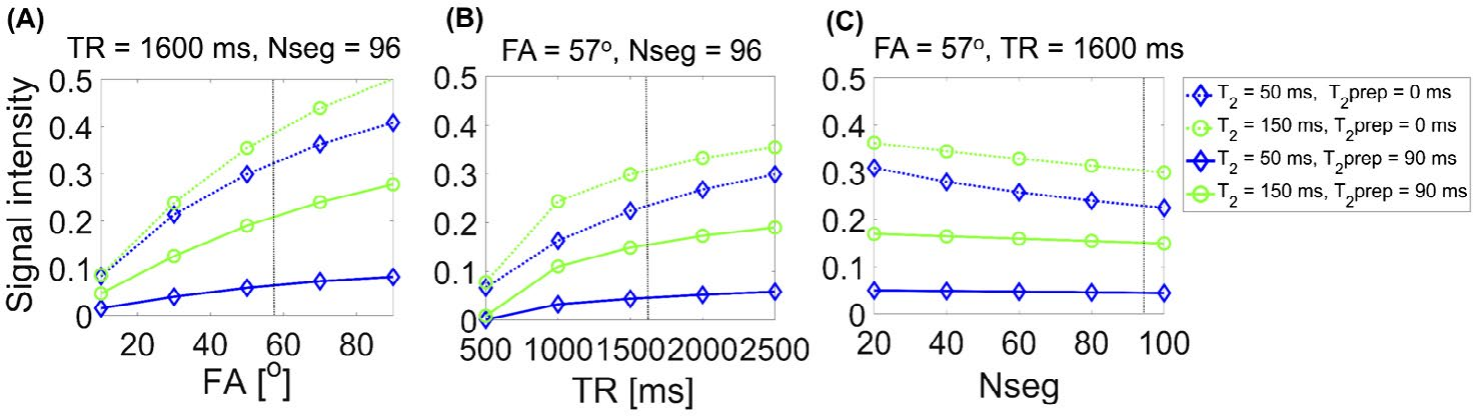
Simulated signal intensity as a function of acquisition parameters FA (A), TR (B), and N_seg_ (C). The signal intensity for each parameter value was obtained from the magnetization response corresponding to T_2_prep duration = 0 ms (dotted lines) or T_2_prep duration = 90 ms (solid lines). The tissues simulated are T_1_^blue^ = 1700 ms, T_2_^blue^ = 50 ms (blue lines), and T_1_^green^ = 1700 ms, T_2_^green^ = 150 ms (green lines)

Image acquisition parameters of the fully sampled and accelerated T_2_prep‐bSSFP sequences were optimized to achieve the best balance between SNR, contrast‐to‐noise ratio (CNR), and a clinically feasible scan time for isotropic 1 mm^3^ 3D T_2_w imaging of the prostate with a T_2_prep TE of 90 ms. Figure 1A‐C illustrates the intensity of the magnetization signal generated with the extended phase graph simulation framework for 2 different T_2_ values as a function of FA, TR, and N_seg_, respectively. The 2 selected T_2_ values (50 ms, 150 ms) represent the average reported values for cancerous and healthy tissue in the peripheral zone of the prostate at 3T.^29^, ^30^, ^31^ As expected, signal intensity increases with increasing shot‐length (TR) as it allows for more time or signal recovery; however, as a trade‐off this will also increase the total acquisition time. Higher FA in the bSSFP readout yields higher SNR. However, due to SAR constraints, a maximum FA of 57° (for a chosen shot‐length of 1600 ms) was achieved.

**Figure 2:**
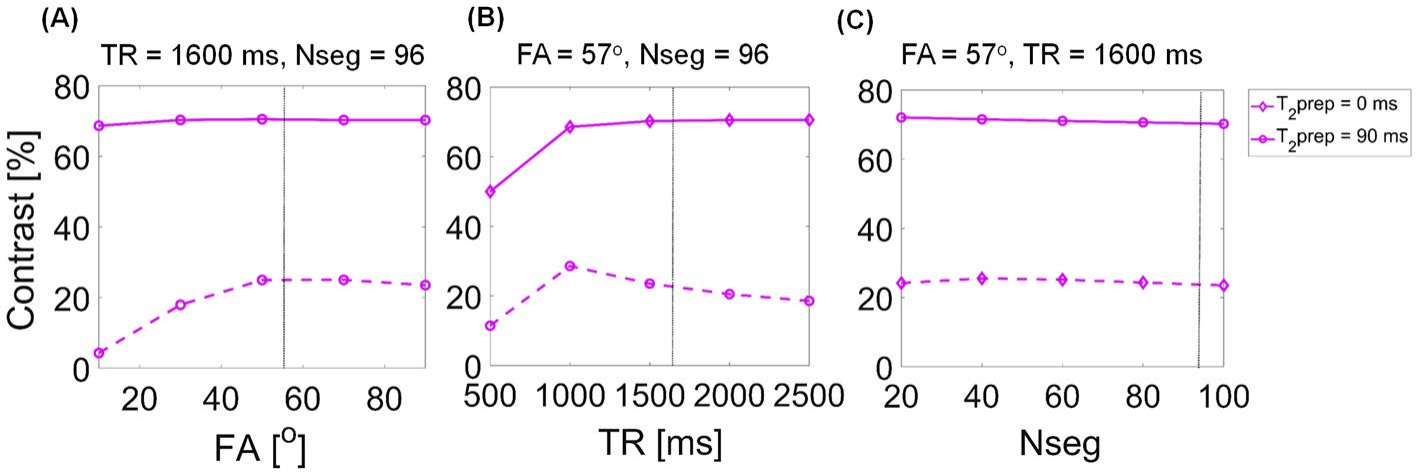
Simulated contrast between 2 representative tissues (healthy and cancerous prostate peripheral zone) as a function of acquisition parameters FA (A), TR (B), and N_seg_ (C)

Importantly, the contrast at a T_2_‐prep duration of 90 ms was found to not change significantly over the simulated range of TR and FA values, as depicted in Figure 2A‐C. It was also observed that increasing the number of segments per shot from 40 to 96 at a T_2_‐prep duration of 90 ms did not cause a large drop in the signal (only 9.6% less signal, Figure 1C) or have an effect on the tissue contrast (only 0.7% less contrast, Figure 2C). But using more segments per shot (96 segments versus 40 segments) reduces the acquisition time by more than half, as fewer shots (spirals) are required to fill‐up k‐space. Guided by these observations, we were able to make the best compromise between the signal intensity, tissue contrast, and acquisition time for in vivo imaging, leading to the following set of optimal sequence parameters for the T_2_prep‐bSSFP sequence: shot‐length^opt^ = 1600 ms, FA^opt^ = 57°, N_seg_^opt^ = 96. The corresponding scan time (min:s) with the optimal parameters for a fully sampled 1 mm^3^ isotropic T_2_w dataset was 8:23, and with the ~3× accelerated T_2_prep‐bSSFP sequence 2:45. In addition, the T_2_prep‐bSSFP signal was verified in a volunteer dataset with the region of interest (ROI) positioned over the central gland of the prostate (Supporting Information Figure S1, which is available online) There is good agreement between the simulated (T_2_fix = 55 ms, T_1_fix = 2200 ms) and measured signal for T_2_prep values (0, 45, 90) ms and TR values (550, 1000, 1500) ms.

### 3.2 In vivo imaging

**Figure 3:**
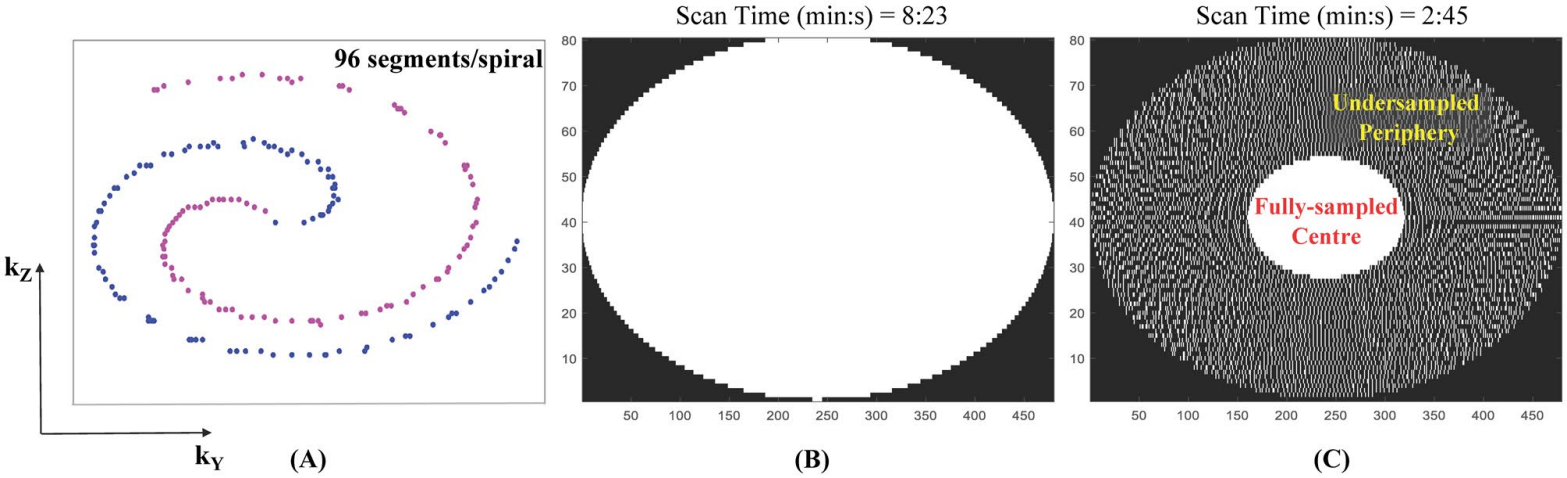
A, Cartesian spiral‐like sampling of the 3D centric CASPR trajectory. The fully sampled and 3× accelerated masks are shown in (B) and (C), respectively

**Figure 4:**
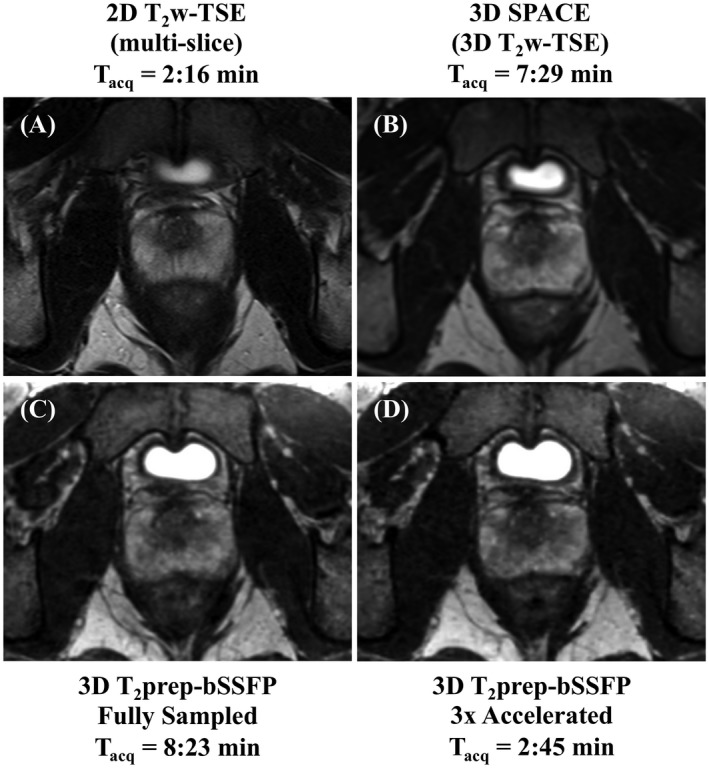
Sequence comparison for a representative healthy subject. A single matched transversal slice was acquired (T_acq_ = acquisition time) using the clinical standard transversal 2D T_2_w‐TSE (TE = 89 ms, 0.6 × 0.8 × 3 mm^3^) (A), 3D SPACE (TE = 101 ms, 1 mm^3^) (B), fully sampled 3D T_2_prep‐bSSFP (TE = 90 ms, 1 mm^3^) (C), and 3× accelerated 3D T_2_prep‐bSSFP (TE = 90 ms, 1 mm^3^) (D) sequences. The accelerated T_2_prep‐bSSFP sequence provides similar image quality compared with the fully sampled reference, and also shows comparable image quality to the clinical standard TSE sequences

Figure 3A shows the acquisition trajectory using Cartesian spirals (shots) that are centric in the k_y_‐k_z_ plane. The fully sampled and undersampled masks are depicted in Figure 3B and C, respectively. The center of k‐space is fully sampled while the periphery is 4‐fold undersampled, resulting in a net acceleration factor of 3×. To illustrate image quality, Figure 4 shows a single matched transversal slice for a representative healthy volunteer scanned using the multislice 2D T_2_w TSE, 3D SPACE, 3D fully sampled T_2_prep‐bSSFP, and 3D accelerated T_2_prep‐bSSFP sequences. It can be observed in Figure 4 (and Supporting Information Figure S2 showing additional subjects) that the accelerated 3D T_2_prep‐bSSFP images have image quality that is comparable to the fully sampled 3D T_2_prep‐bSSFP and clinical standard high resolution 2D T_2_w TSE images, and appear visually sharper than the 1 mm^3^ isotropic 3D SPACE images.

**Figure 5:**
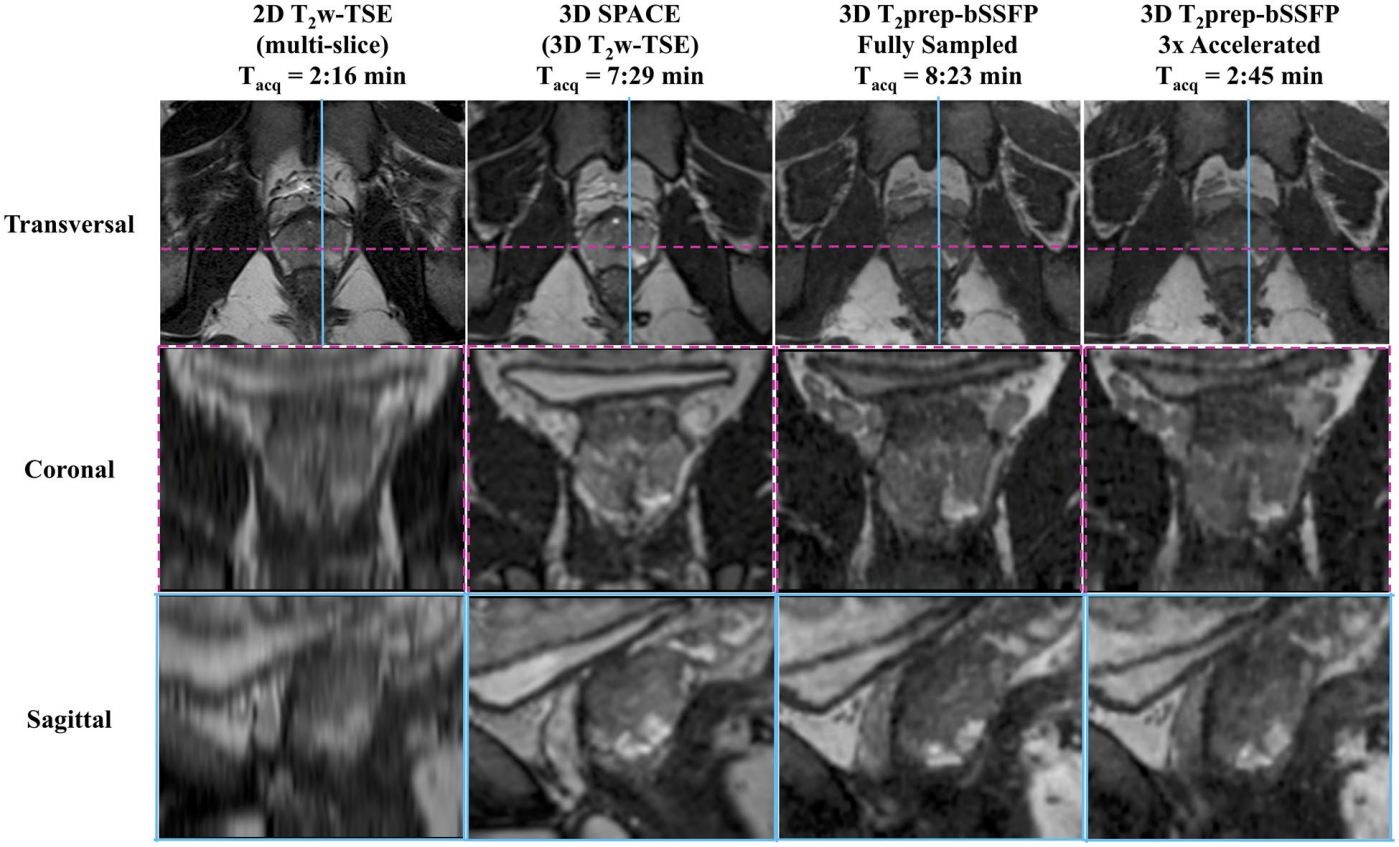
A comparison of different imaging planes from a representative healthy subject with 1‐mm isotropic acquisition (T_acq_ = acquisition time). A single matched transversal slice was reformatted into the sagittal and coronal planes for the multislice 2D T_2_w‐TSE, 3D SPACE, fully sampled and accelerated 3D T_2_prep‐bSSFP sequences. The coronal and sagittal reformats corresponding to the 2D T_2_w‐TSE have poor image quality due to the multislice 2D transversal acquisition and 0.6 × 0.8 × 3 mm^3^ resolution. The 3× accelerated T_2_prep‐bSSFP images show comparable image quality particularly with the other 3D sequences in all 3 orientations

Figure 5 and Supporting Information Figure S3 illustrate a single matched transversal slice for 2 representative healthy subjects from the multislice 2D T_2_w TSE, 3D SPACE, 3D fully sampled and accelerated T_2_prep‐bSSFP reconstructions, reformatted into the coronal and sagittal planes to facilitate comparisons in all 3 imaging orientations. The poor image quality of the coronal and sagittal reformats corresponding to the 2D T_2_w TSE can be attributed to the multislice 2D transversal acquisition and low resolution along the slice dimension (0.6 × 0.8 × 3 mm^3^). The accelerated T_2_prep‐bSSFP acquisitions appear to have no visually remaining artifacts from the TV‐SENSE reconstruction along all the 3 dimensions, and display comparable image quality with the fully sampled T_2_prep‐bSSFP and clinical standard TSE sequences. The normalized root mean square error between the fully sampled and 3× accelerated T_2_prep‐bSSFP reconstructions for the volunteer group was (1.6 ± 0.46) %. The mean “apparent SNR” and normalized contrast comparisons (tissue/noise ROIs chosen are shown in Supporting Information Figure S4) between the sequences are shown in Supporting Information Table S1 and Supporting Information Table S2, respectively. The fully sampled and 3× accelerated T_2_prep‐bSSFP images, which were full field of view (FOV) acquisitions with matched imaging parameters, show comparable “apparent SNR” and contrast for the whole prostate, muscle, and fat.

### 3.3 Evaluation of image quality

**Figure 6:**
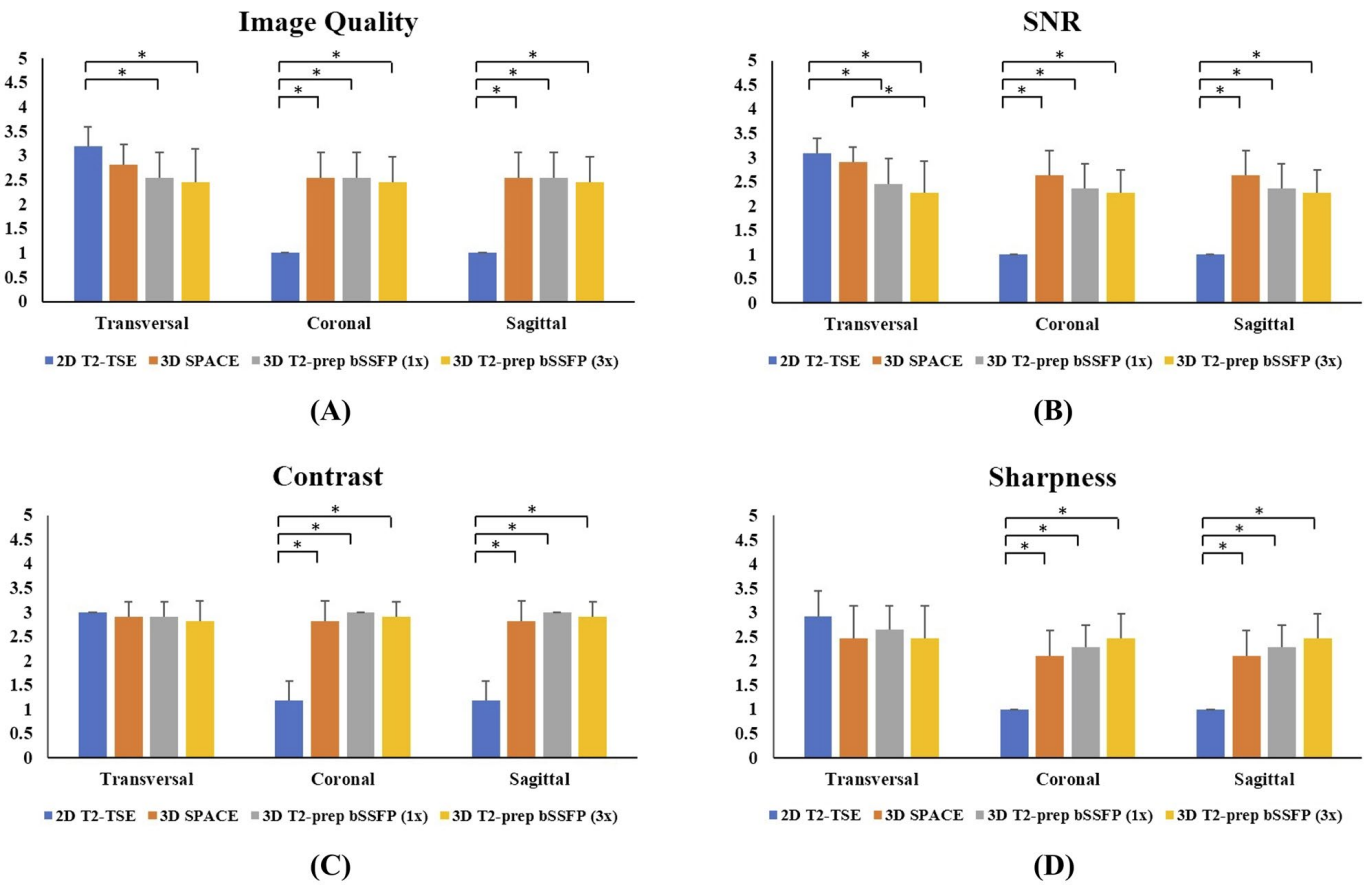
A qualitative/subjective evaluation of the image quality (A), SNR (B), tissue contrast (C), and image sharpness (D) in the overall prostate by 2 experienced readers. Results depict the combined “agreed score” assigned by the 2 readers for each image quality metric according to the scoring criteria listed in Table 2. The non‐diagnostic outliers are from the multislice 2D T_2_w TSE reformats along the coronal and sagittal orientations. (*P < 0.05)

Figure 6 summarizes the expert reader image quality scoring of the accelerated T_2_prep‐bSSFP reconstructions, in comparison with the fully sampled T_2_prep‐bSSFP and clinical standard 2D and 3D (SPACE) T_2_w‐TSE images. In the transversal slices, the 3× accelerated T_2_prep‐bSSFP images had very similar contrast (2.82 ± 0.41) and image sharpness (2.46 ± 0.69), with no statistically significant differences (*P* > 0.05) seen among any of the 4 sequences under consideration, but lower subjective SNR (2.27 ± 0.65; *P* < 0.05) as compared to the other 3 sequences. The multislice 2D T_2_w TSE images (with higher in‐plane resolution 0.6 × 0.8 mm^2^ and thicker slices of 3 mm) had the best overall image quality in the prostate in the transversal orientation, scoring better (*P* < 0.05) than the 3D T_2_prep‐bSSFP images (with 1 mm^3^ isotropic resolution). However, all three 3D sequences were found to be very comparable (*P* > 0.05) in terms of the overall image quality in the prostate.

Similar scores and trends were observed for the accelerated T_2_prep‐bSSFP reformats along the coronal and sagittal slices (Figure 6). As expected, the 2D T_2_w‐TSE was found to have non‐diagnostic image quality metrics along the coronal and sagittal reformats due to the multislice acquisition and anisotropic image resolution of 0.6 × 0.8 × 3 mm^3^. In this study, it would not be fair to compare the reformats from the multislice 2D T_2_w‐TSE with those from the true 3D acquisitions as the clinician would typically be evaluating the high resolution T_2_w images acquired individually along the coronal and sagittal orientations. The images from all three 3D sequences were found to have very similar (*P* > 0.05) image quality metrics along the coronal and sagittal orientations, as illustrated in Figure 6A‐D. Some visually apparent banding and residual reconstruction artifacts (absent in the clinical 2D and 3D T_2_w‐TSE sequences) were noted by the experts in the fully sampled and accelerated T_2_prep‐bSSFP images for a few volunteers. However, these artifacts were external to the prostate and were found to not affect the diagnostic quality of the images.

## 4 DISCUSSION

Our results show that 3D multishot T_2_prep‐bSSFP acquisitions with variable density undersampling combined with TV‐SENSE reconstruction enable rapid T_2_w imaging of the whole prostate with 1‐mm isotropic spatial resolution in under 3 min at 3T. The accelerated T_2_prep‐bSSFP images display similar image quality to the fully sampled T_2_prep‐bSSFP and conventional clinical standard 2D/3D T_2_w TSE images, with the clear advantage of requiring only one‐third the acquisition time. Our technique does not suffer from geometric distortion as it is gradient echo based, and is thus ideally suited for MR‐guided radiotherapy and artificial intelligence (AI) applications that depend on high resolution data. Our approach is also suitable for fast 3D quantitative T_2_ mapping^32^ (that requires using multiple different T_2_‐prep durations) with 1‐mm isotropic resolution, which is currently not included in the clinical protocol for prostate mp‐MRI due to the prohibitively long scan time.

Isotropic high resolution T_2_w imaging of entire anatomical structures in vivo with sufficient volumetric coverage still poses a challenge, mainly due to the long scan time and lack of sufficient SNR. Multiplanar, multislice 2D T_2_w TSE imaging along 3 orthogonal planes is the current gold standard in prostate imaging, requiring a total scan time of over 7‐12 min to acquire all 3 orientations, each having a nonisotropic resolution of ~0.7 × 0.7 × 3 mm^3^.^11^, ^33^ True 3D T_2_w imaging, using a 3D TSE sequence such as the SPACE requires a scan time of over 7 min, as seen in our study and previously.^11^ Importantly, SPACE is usually a zoomed (reduced FOV) acquisition, while our proposed approach offers the advantage of rapid full FOV imaging, which is a requirement in applications like radiotherapy planning. A full‐FOV SPACE acquisition would require a prohibitively long scan time. Acceleration techniques like parallel imaging (R = 2) have been used to reduce the scan time (min:s) of the SPACE sequence to 3:52 for a resolution of 1 × 1.5 × 1.5 mm^3^ with 2 averages,^34^ which is still longer and has a lower spatial resolution than our proposed approach. The 3D SPACE images also tend to appear blurry due to motion and signal decay along the long echo train. Our approach using centric encoded T_2_‐prepared 3D bSSFP imaging is not affected by blurring as the center of k‐space is acquired at the beginning of the shot, and immediately after T_2_ preparation. As each shot starts with acquiring data in a central region of k‐space using a Cartesian “spiral‐out” trajectory, motion will be averaged over multiple shots.

Rapid 3D T_2_w imaging in the prostate has been recently investigated using the T_2_‐transition into driven equilibrium (T_2_‐TIDE) and dual echo steady state (DESS) sequences.^13^, ^14^ A 3D T_2_‐TIDE sequence was used by Srinivasan et al^14^ for fast T_2_w imaging of the entire prostate in 2:54 at a spatial resolution of 0.9 × 0.8 × 1.5 mm^3^, with 2 averages and no additional acceleration methods. While this technique has comparable image quality to and is ~3× faster than the standard 3D TSE, the accelerated T_2_prep‐bSSFP acquisitions achieve a higher spatial resolution along the slice dimension for the same acquisition duration. Dregely et al^13^ used the DESS sequence for rapid 3D (1.1 × 1.1 × 3.5 mm^3^) T_2_w prostate imaging using 1 average and no additional acceleration schemes in a scan time (min:s) of 1:03. In comparison, our accelerated T_2_prep‐bSSFP acquisitions can achieve the same image resolution in 37 s.

Our approach required an optimization of the T_2_prep‐bSSFP sequence parameters to achieve the best balance between the SNR, CNR, and scan time. A shot‐length = 1600 ms and FA = 57° was chosen based on the results from our extended phase graph simulations, keeping in mind the long prostate T_1_ recovery times and SAR considerations at 3T. In particular, the increased number of readout segments (N_seg_ = 96), compared with the previously used 40 segments^17^, ^35^ translates in a decreased number of shots (Cartesian spirals) required to fill the k‐space, and thus in a significant scan time reduction (58%) while maintaining a comparable SNR and CNR. The TV‐SENSE reconstruction allowed the generation of artifact‐free images of the 3× accelerated T_2_prep‐bSSFP acquisitions, which were 4× undersampled in the peripheral k‐space.

The accelerated T_2_prep‐bSSFP images were perceived by the 2 readers to have comparable image quality to the fully sampled T_2_prep‐bSSFP and clinical standard TSE sequences, particularly preserving high tissue contrast and image sharpness along all 3 dimensions. The T_2_ contrast depends on the TE, TR, T_1_, and T_2_ of the tissues under consideration, B_0_, and B_1_ effects. In the 2D and 3D T_2_w TSE sequences, contribution from stimulated and indirect echoes in the multiecho signal affects the final T_2_ contrast. We have previously evaluated the effect of B_1_/T_1_ variations on the T_2_ contrast of the T_2_prep‐bSSFP sequence.^32^ The Cartesian spiral‐out acquisition in the k_y_‐k_z_ plane enables immediate encoding of the magnetization prepared signal, thus preserving the T_2_ weighting. The 3× accelerated T_2_prep‐bSSFP acquisitions did not suffer from loss in T_2_ contrast as the center of k‐space (25%) was fully sampled. The lower subjective SNR of the accelerated T_2_prep‐bSSFP images was found to not limit the overall diagnostic quality of the whole prostate. In the cases where the fully sampled T_2_prep‐bSSFP images scored lower than the 3× accelerated ones, the better performance of the accelerated sequence could potentially be attributed to the faster acquisition reducing the susceptibility to any motion artifacts, and the effect of additional de‐noising from the TV‐SENSE reconstruction.

In this study, the read‐out was along the anterior‐to‐posterior direction to reduce susceptibility to motion artifacts. Setting the read‐out along the right‐to‐left direction can further reduce the acquisition time as the FOV in the right‐to‐left direction is ~2× the FOV in the anterior‐to‐posterior direction. However, acquisitions along the anterior‐to‐posterior direction are more prone to motion and might require additional motion compensation schemes. For example, 2D image navigators acquired during the bSSFP catalyzation period can be incorporated in the reconstruction for retrospective motion correction. Further acceleration could potentially be achieved using techniques like multidimensional parallel imaging, view sharing, and improved design of the k‐space trajectory (e.g., a center oversampled trajectory to improve the SNR). However, there is a trade‐off between acceleration and SNR with increasing sparsity, particularly in pure 3D high resolution isotropic imaging, which needs to be investigated.

Our study has several limitations. First, the undersampled data had to be reconstructed offline in Matlab^TM^ using TV‐SENSE, the time for reconstruction being ~20 min for each 3× undersampled 3D T_2_w dataset. Second, both the fully sampled and accelerated T_2_prep‐bSSFP acquisitions are susceptible to banding artifacts arising from off‐resonance effects. While artifacts were not observed over the prostate region in the healthy volunteers, slight banding was observed in the FOV in areas away from the ROIs in a few subjects. The standard shim was adopted for the healthy volunteer study following optimization of the protocol settings, and could require further optimization in patients. Our future approach would also involve using a gradient recalled echo readout to eliminate banding. Third, a true quantitative assessment of the SNR between the sequences under consideration was not feasible as there is no agreed method for SNR evaluation in the literature for regularized reconstructions. Finally, we have evaluated the feasibility of our accelerated T_2_prep‐bSSFP acquisitions in a healthy volunteer cohort. Future studies would involve testing our proposed approach in prostate cancer patients to establish its utility over the current clinical standard TSE sequences.

Considering the above factors, clinically the proposed approach may be best suited to substitute at least one of the multiplanar 2D T_2_w TSE acquisitions, serving as a partial rather than absolute replacement for conventional multiplanar T_2_w imaging. The high‐resolution isovolumetric images obtained in an acceptable time frame provide a better model for image‐fusion co‐registration, which is increasingly being used for biopsy and focal therapy in prostate MRI.

## 5 CONCLUSIONS

In conclusion, we have demonstrated the feasibility of 3D T_2_w imaging in the prostate with 1‐mm isotropic resolution in 2:45 min, by combining 3D multishot T_2_prep‐bSSFP acquisitions with a variable density undersampled trajectory and TV‐SENSE reconstruction. This facilitates high‐resolution isotropic prostate imaging in a clinically acceptable scan time.

## CONFLICTS OF INTEREST

Dr. Radhouene Neji is an employee of Siemens Healthcare Limited. Prof. Vicky Goh receives research support from Siemens Healthcare Limited.

## Supporting information


**FIGURE S1** A, Verification of the T_2_prep‐bSSFP signal in a volunteer with the ROI positioned over the central gland of the prostate. Comparison between the simulated and measured signal for: T_2_ values (40:5:80) ms (B), T_2_prep values (0, 45, 90) ms with T_2_fix = 55 ms (C), and TR values (550, 1000, 1500) ms with T_2_fix = 55 ms (D). The T_1_ was fixed at 2200 ms for the simulations. Error bars indicate the standard deviation of the signal within the ROI
**FIGURE S2** Sequence comparison for 5 representative healthy subjects. A single matched transversal slice acquired (T_acq_ = acquisition time) in each case using the clinical standard transversal 2D T_2_w‐TSE (TE = 89 ms, 0.6 × 0.8 × 3 mm^3^) (A), 3D SPACE (TE = 101 ms, 1 mm^3^) (B), fully sampled 3D T_2_prep‐bSSFP (TE = 90 ms, 1 mm^3^) (C), and 3× accelerated 3D T_2_prep‐bSSFP (TE = 90 ms, 1 mm^3^) (D) sequences
**FIGURE S3** A comparison of different imaging planes from a representative healthy subject with 1‐mm isotropic acquisition (T_acq_ = acquisition time). A single matched transversal slice was reformatted into the sagittal and coronal planes for the multislice 2D T_2_w‐TSE, 3D SPACE, fully sampled and accelerated 3D T_2_prep‐bSSFP scans. The coronal and sagittal reformats corresponding to the 2D T_2_w‐TSE have poor image quality due to the multislice 2D transversal acquisition and 0.6 × 0.8 × 3 mm^3^ resolution
**FIGURE S4** ROIs selected for the whole prostate, muscle, fat, and background noise to compute the “apparent SNR” and normalized contrast in Supporting Information Table S1 and Supporting Information Table S2, respectively
**TABLE S1** Comparison of the “apparent SNR,” computed using the standard ROI method, in the whole prostate, muscle, and fat for the fully sampled and 3× accelerated 3D T_2_prep‐bSSFP acquisitions. The ROIs for the different tissues/noise are shown in Supporting Information Figure S4. In addition, the SNR is not reported for the clinical standard 2D and 3D T_2_w TSE sequences as these acquisitions were not full FOV and zoomed to the prostate, and thus did not have a “noise ROI”
**TABLE S2** Contrast comparisons between the whole prostate, muscle, and fat for the 4 sequences under consideration. The normalized contrast difference between the tissues was calculated as (SI_tissue1_ − SI_tissue2_)/ (SI_tissue1_ + SI_tissue2_), where SI is the mean signal intensity. ROIs for the different tissues are shown in Supporting Information Figure S4. Furthermore, (1) fat suppression was off for all sequences, (2) the acquired resolution and averages are not the same across all the techniques as the reference sequences were acquired according to PI‐RADS specifications, (3) the 2D and 3D T_2_w TSE images were directly extracted from the scanner and generated using a vendor‐specific black‐box reconstruction and post‐processing pipeline
