# Supplementary material for: Accelerated 3D T2w‐imaging of the prostate with 1‐millimeter isotropic resolution in less than 3 minutes
Source: Magn Reson Med. 2019 Apr 21;82(2):721–31. doi: 10.1002/mrm.27764 (PMC6563534; doi:10.1002/mrm.27764)
Supplement: Supplementary file 1 — FIGURE S1 A, Verification of the T2prep‐bSSFP signal in a volunteer with the ROI positioned over the central gland of the prostate. Comparison between the simulated and measured signal for: T2 values (40:5:80) ms (B), T2prep values (0, 45, 90) ms with T2fix = 55 ms (C), and TR values (550, 1000, 1500) ms with T2fix = 55 ms (D). The T1 was fixed at 2200 ms for the simulations. Error bars indicate the standard deviation of the signal within the ROI FIGURE S2 Sequence comparison for 5 representative healthy subjects. A single matched transversal slice acquired (Tacq = acquisition time) in each case using the clinical standard transversal 2D T2w‐TSE (TE = 89 ms, 0.6 × 0.8 × 3 mm3) (A), 3D SPACE (TE = 101 ms, 1 mm3) (B), fully sampled 3D T2prep‐bSSFP (TE = 90 ms, 1 mm3) (C), and 3× accelerated 3D T2prep‐bSSFP (TE = 90 ms, 1 mm3) (D) sequences FIGURE S3 A comparison of different imaging planes from a representative healthy subject with 1‐mm isotropic acquisition (Tacq = acquisition time). A single matched transversal slice was reformatted into the sagittal and coronal planes for the multislice 2D T2w‐TSE, 3D SPACE, fully sampled and accelerated 3D T2prep‐bSSFP scans. The coronal and sagittal reformats corresponding to the 2D T2w‐TSE have poor image quality due to the multislice 2D transversal acquisition and 0.6 × 0.8 × 3 mm3 resolution FIGURE S4 ROIs selected for the whole prostate, muscle, fat, and background noise to compute the “apparent SNR” and normalized contrast in Supporting Information Table S1 and Supporting Information Table S2, respectively TABLE S1 Comparison of the “apparent SNR,” computed using the standard ROI method, in the whole prostate, muscle, and fat for the fully sampled and 3× accelerated 3D T2prep‐bSSFP acquisitions. The ROIs for the different tissues/noise are shown in Supporting Information Figure S4. In addition, the SNR is not reported for the clinical standard 2D and 3D T2w TSE sequences as these acquisitions were not full FOV and zoomed to [file MRM-82-721-s001.docx]

**Supporting Information**

**
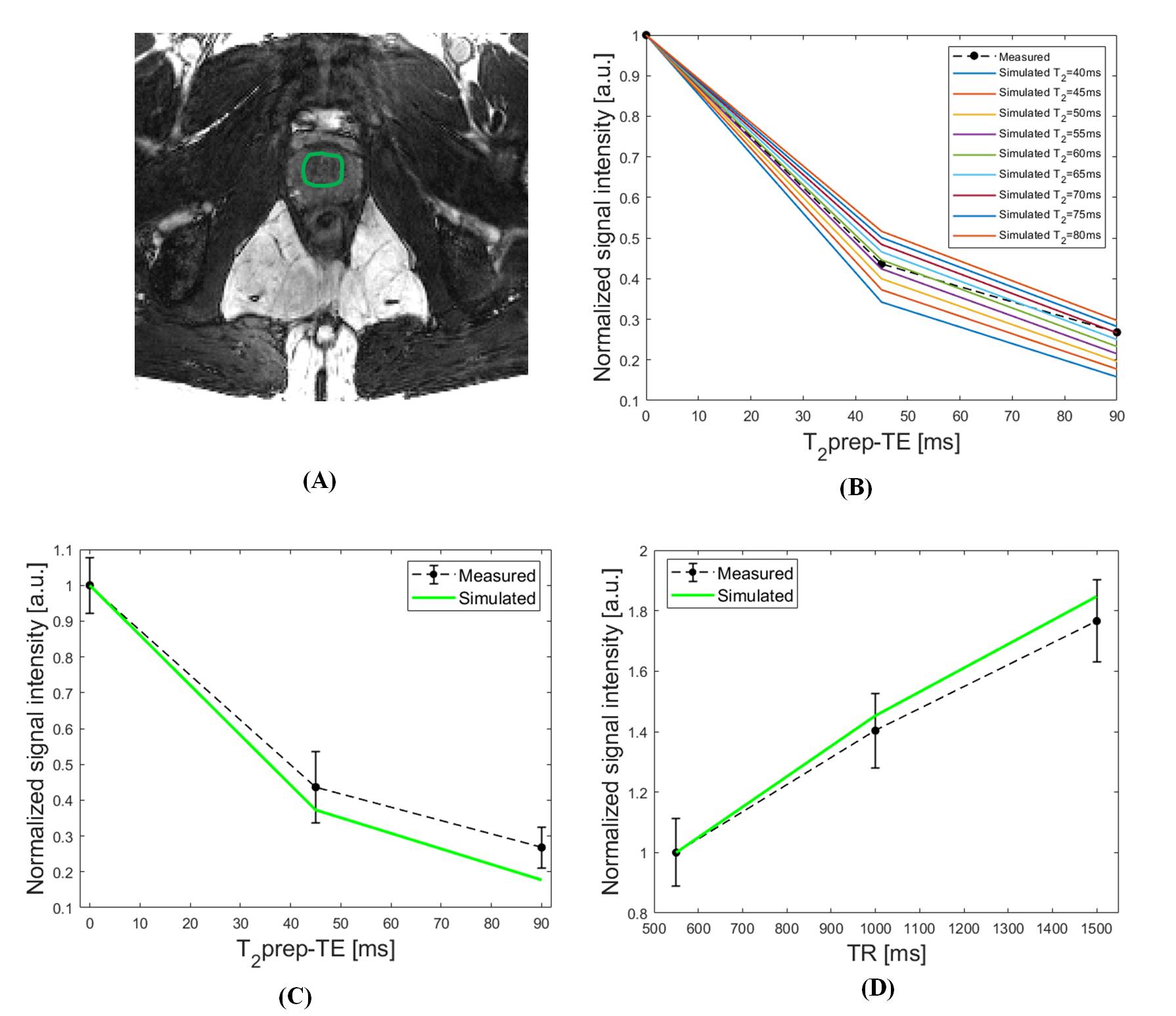
**

**Supporting Information Figure S1:** Verification of the T_2_prep-bSSFP signal in a volunteer with the region of interest (ROI) positioned over the central gland of the prostate in (A). Comparison between the simulated and measured signal for: (B) T_2_ values (40:5:80) ms, (C) T_2_prep values (0, 45, 90) ms with T_2_fix = 55 ms, and (D) TR values (550, 1000, 1500) ms with T_2_fix = 55ms. The T_1_ was fixed at 2200 ms for the simulations. Error bars indicate the standard deviation of the signal within the ROI.

**
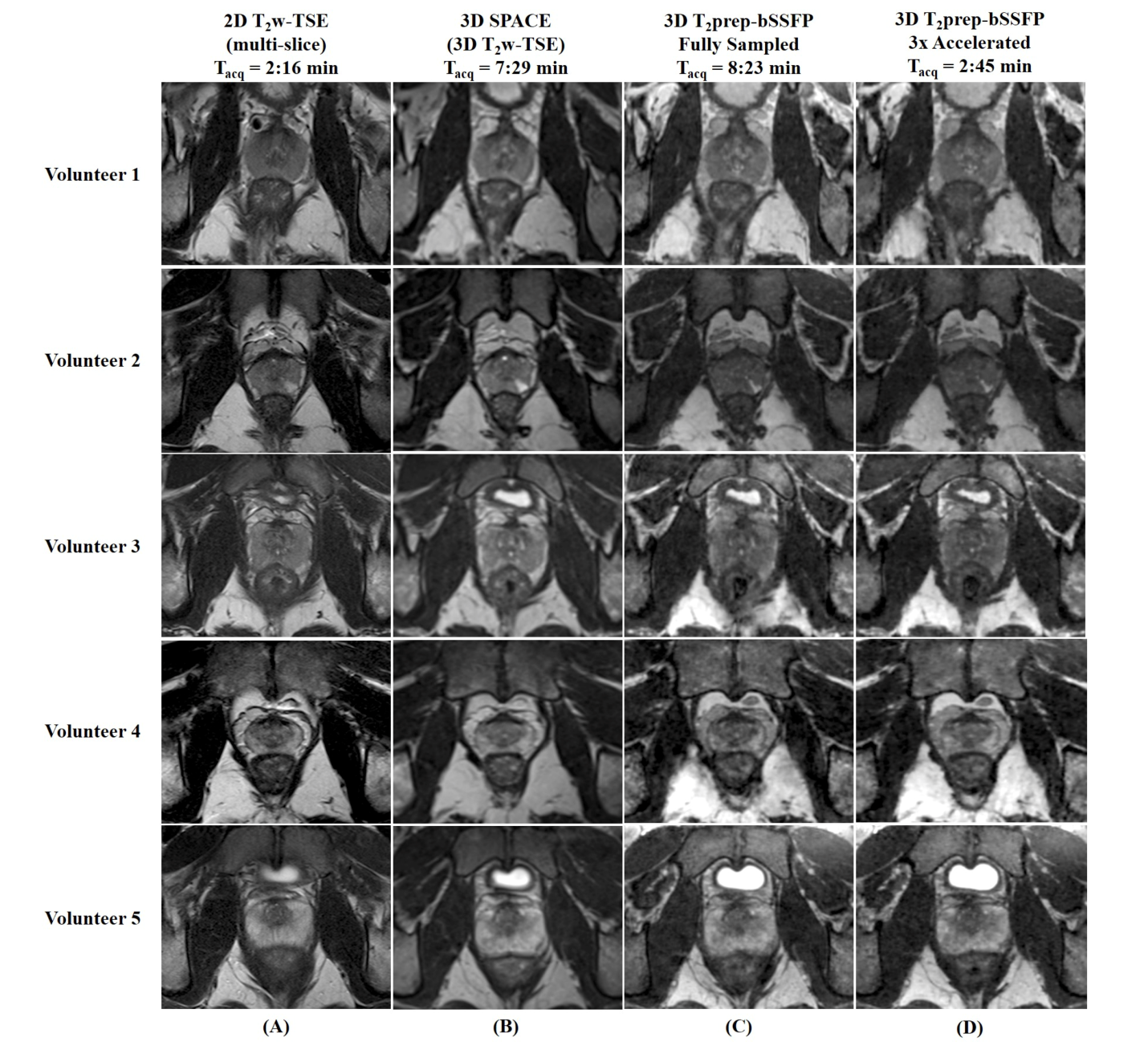
**

**Supporting Information Figure S2:** Sequence comparison for five representative healthy subjects. A single matched transversal slice acquired (T_acq_ = acquisition time) in each case using the (A) clinical standard transversal 2D T_2_w-TSE (TE = 89 ms, 0.6 x 0.8 x 3 mm^3^), (B) 3D SPACE (TE = 101 ms, 1 mm^3^), (C) fully-sampled 3D T_2_prep-bSSFP (TE = 90 ms, 1 mm^3^), and (D) 3x accelerated 3D T_2_prep-bSSFP (TE = 90 ms, 1 mm^3^) sequences.

**
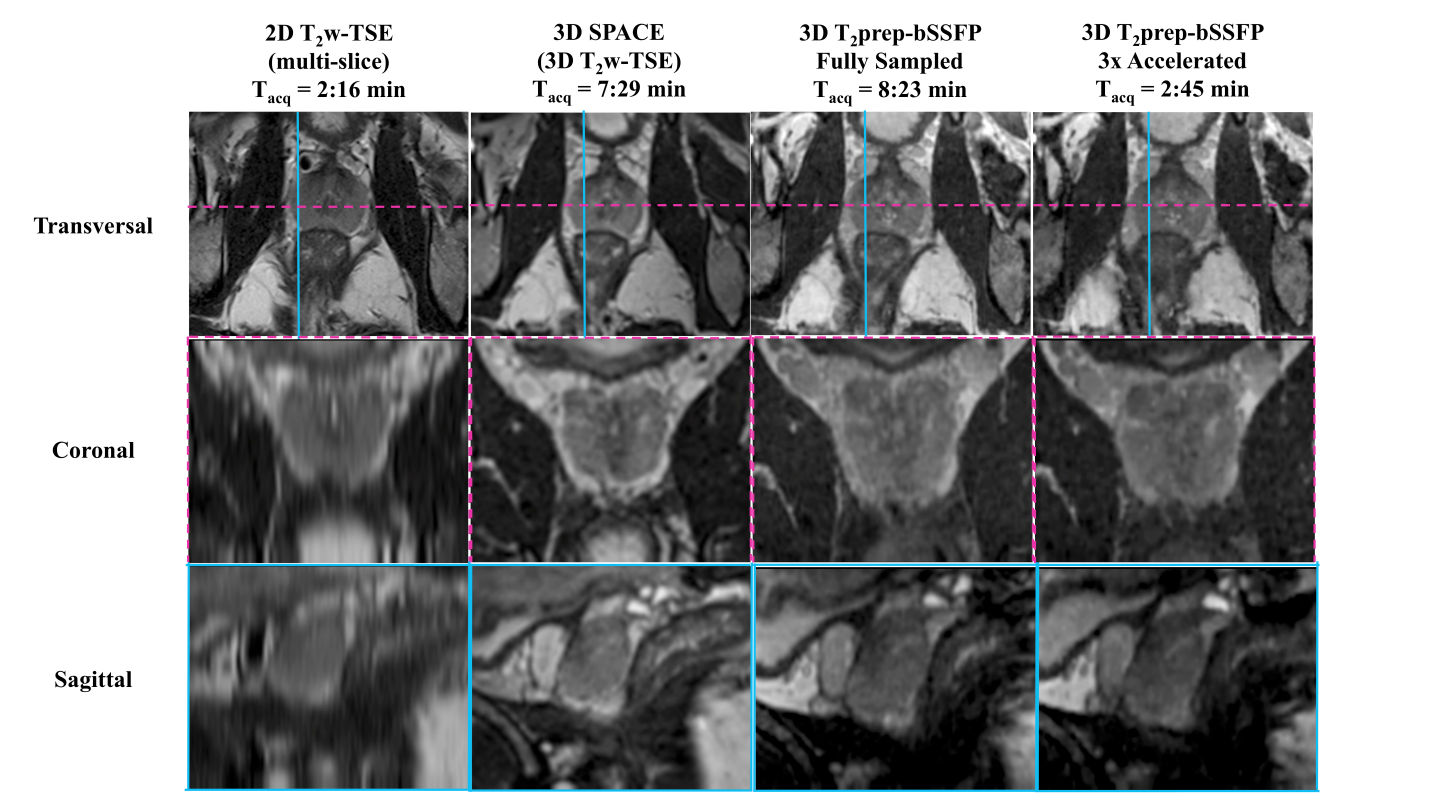
**

**Supporting Information Figure S3:** A comparison of different imaging planes from a representative healthy subject with 1 mm isotropic acquisition (T_acq_ = acquisition time). A single matched transversal slice was reformatted into the sagittal and coronal planes for the multi-slice 2D T_2_w-TSE, 3D SPACE, fully-sampled and accelerated 3D T_2_prep-bSSFP scans. The coronal and sagittal reformats corresponding to the 2D T_2_w-TSE have poor image quality due to the multi-slice 2D transversal acquisition and 0.6 x 0.8 x 3 mm^3^ resolution.


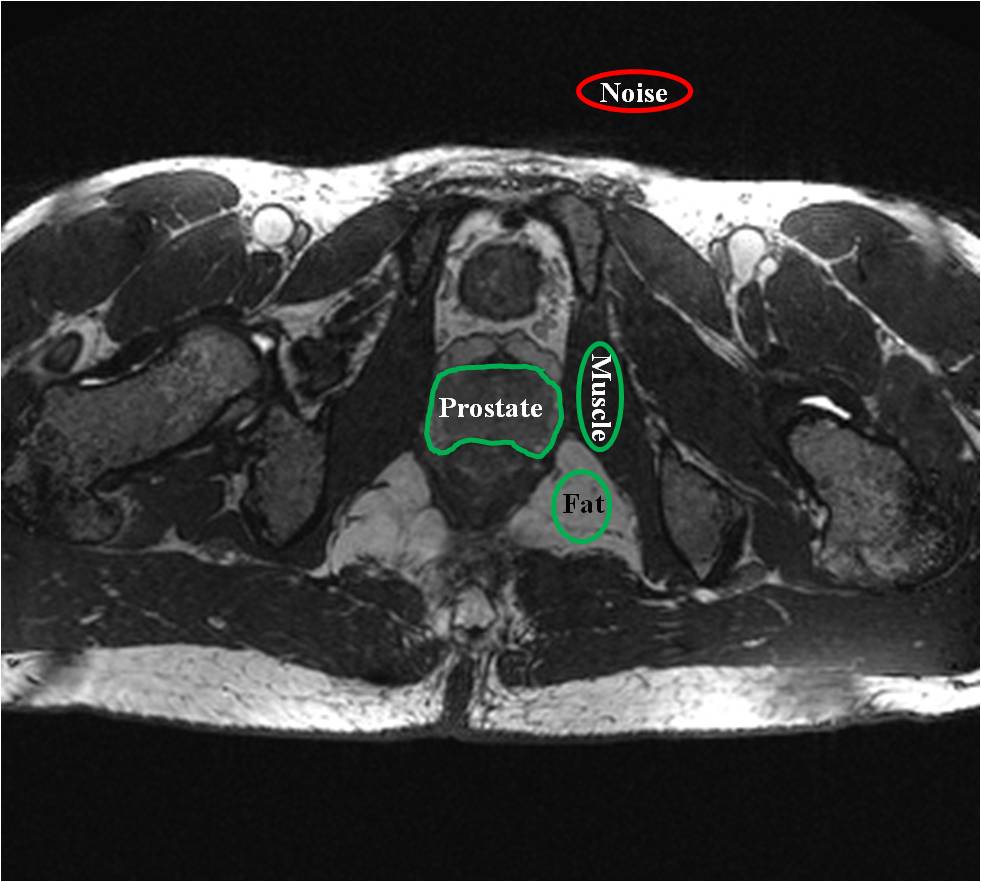


**Supporting Information Figure S4:** Regions of Interest (ROIs) selected for the whole prostate, muscle, fat, and background noise to compute the ‘apparent SNR’ and normalized contrast in Supporting Information Table S1 and Supporting Information Table S2, respectively.

**Supporting Information Table S1**: Comparison of the ‘apparent SNR’, computed using the standard Region of Interest (ROI) method, in the whole prostate, muscle, and fat for the fully-sampled and 3X accelerated 3D T_2_prep-bSSFP acquisitions. The ROIs for the different tissues/noise are shown in Supporting Information Figure S4. In addition, the SNR is not reported for the clinical standard 2D and 3D T_2_w TSE sequences as these acquisitions were not full FOV and zoomed to the prostate, and thus did not have a ‘noise ROI’.

| **Sequence** | **Whole Prostate** | **Fat** | **Muscle** |
| --- | --- | --- | --- |
| T_2_prep-bSSFP (1X) | 23.8 ± 3.2 | 57.8 ± 10.5 | 8.9 ± 0.8 |
| T_2_prep-bSSFP (3X) | 21.9 ± 5.6 | 50.3 ± 13.6 | 8.2 ± 1.9 |

**Supporting Information Table S2**: Contrast comparisons between the whole prostate, muscle, and fat for the four sequences under consideration. The normalized contrast difference between the tissues was calculated as (SI_tissue1_ - SI_tissue2_)/ (SI_tissue1_ + SI_tissue2_), where SI is the mean signal intensity. Regions of Interest (ROIs) for the different tissues are shown in Supporting Information Figure S4. Furthermore, 1) fat suppression was off for all sequences, 2) the acquired resolution and averages are not the same across all the techniques as the reference sequences were acquired according to PI-RADS specifications, 3) the 2D and 3D T_2_w TSE images were directly extracted from the scanner and generated using a vendor-specific black-box reconstruction and post-processing pipeline.

| **Sequence** | **Normalized Contrast** | | | **Acquired Resolution (mm^3^)** | **Number of Averages** |
| --- | --- | --- | --- | --- | --- |
|  | **Prostate & Muscle** | **Prostate & Fat** | **Muscle & Fat** |  |  |
| 2D T_2_w TSE | 0.66 ± 0.04 | 0.31 ± 0.07 | 0.8 ± 0.03 | 0.6 x 0.8 x 3 | 2 |
| 3D T_2_w TSE | 0.69 ± 0.05 | 0.22 ± 0.08 | 0.78 ± 0.05 | 1 x 1 x 1 | 2 |
| T_2_prep-bSSFP (1X) | 0.46 ± 0.06 | 0.41 ± 0.09 | 0.73 ± 0.03 | 1 x 1 x 1 | 1 |
| T_2_prep-bSSFP (3X) | 0.46 ± 0.05 | 0.39 ± 0.09 | 0.72 ± 0.05 | 1 x 1 x 1 | 1 |
